# Content-Based Recommender Support System for Counselors in a Suicide Prevention Chat Helpline: Design and Evaluation Study

**DOI:** 10.2196/21690

**Published:** 2021-01-07

**Authors:** Salim Salmi, Saskia Mérelle, Renske Gilissen, Willem-Paul Brinkman

**Affiliations:** 1 Department of Stochastics Centrum Wiskunde & Informatica Amsterdam Netherlands; 2 113 Suicide Prevention Amsterdam Netherlands; 3 Interactive Intelligence Delft University of Technology Delft Netherlands

**Keywords:** suicide prevention, content based recommender system, chat corpus, crisis line, sentence embedding, suicide, mental health

## Abstract

**Background:**

The working environment of a suicide prevention helpline requires high emotional and cognitive awareness from chat counselors. A shared opinion among counselors is that as a chat conversation becomes more difficult, it takes more effort and a longer amount of time to compose a response, which, in turn, can lead to writer’s block.

**Objective:**

This study evaluates and then designs supportive technology to determine if a support system that provides inspiration can help counselors resolve writer’s block when they encounter difficult situations in chats with help-seekers.

**Methods:**

A content-based recommender system with sentence embedding was used to search a chat corpus for similar chat situations. The system showed a counselor the most similar parts of former chat conversations so that the counselor would be able to use approaches previously taken by their colleagues as inspiration. In a within-subject experiment, counselors’ chat replies when confronted with a difficult situation were analyzed to determine if experts could see a noticeable difference in chat replies that were obtained in 3 conditions: (1) with the help of the support system, (2) with written advice from a senior counselor, or (3) when receiving no help. In addition, the system’s utility and usability were measured, and the validity of the algorithm was examined.

**Results:**

A total of 24 counselors used a prototype of the support system; the results showed that, by reading chat replies, experts were able to significantly predict if counselors had received help from the support system or from a senior counselor (*P*=.004). Counselors scored the information they received from a senior counselor (M=1.46, SD 1.91) as significantly more helpful than the information received from the support system or when no help was given at all (M=–0.21, SD 2.26). Finally, compared with randomly selected former chat conversations, counselors rated the ones identified by the content-based recommendation system as significantly more similar to their current chats (β=.30, *P*<.001).

**Conclusions:**

Support given to counselors influenced how they responded in difficult conversations. However, the higher utility scores given for the advice from senior counselors seem to indicate that specific actionable instructions are preferred. We expect that these findings will be beneficial for developing a system that can use similar chat situations to generate advice in a descriptive style, hence helping counselors through writer’s block.

## Introduction

Worldwide, helplines have been set up to help individuals who are struggling with suicidal thoughts. These helplines are a preventive service to reduce the suicidal ideation or behavior of help-seekers [1]. These help-seekers can contact trained volunteers and professionals (counselors) who can listen to them and assist them with their problems relating to suicide.

Historically, people have been able to contact these helplines over the telephone, but with the advent of the internet, chat services have become increasingly popular. Compared with telephone helplines, online chat helplines show approximately the same beneficial effects [2]. Help-seekers mention several reasons for using counseling through an online chat rather than a traditional phone call, such as privacy and the slow deliberate nature of online chatting [3-6]. In the Netherlands, the 113 Suicide Prevention service saw the number of conversations increase to more than 35,000 via telephone and more than 57,000 via online chat in 2018, an increase of 33% from 2017. However, this increase resulted in a higher need for counselors as well. Because of the difficult nature of crisis counseling, suicide prevention helplines often have difficulty retaining counselors [7].

Studies have indicated that technology can support chat line operators in executing cognitive tasks. For example, in the related field of commercial telephone and chat customer support, there are various supportive technologies developed for operators [8-10]. However, in computing research aimed at suicide prevention, most work focuses on the prediction and detection of suicidal behavior [11,12], while only a few studies have examined assisting online counselors; this could be beneficial, though. Salmi [13] has identified several difficulties that counselors encounter in their work. First, the counselor has to take in a large amount of information about the help-seeker. Here, counselors could be supported in understanding a help-seeker’s history without having to read large portions of transcripts. Dinakar et al [14], therefore, have created a support system prototype for text-based crisis counseling called Fathom. Fathom uses visualizations based on topic modeling to provide information at a glance. In comparison to a control interface without a visualization aspect, Fathom was preferred by counselors when eliciting a list of issues and a conversation summary. Another difficulty is that the counselor must be aware of the conversation quality. In this respect, Althoff et al [15] compared the chat conversations of more and less successful counselors with natural language processing techniques to discover the quality differences, defining actionable strategies to improve conversation quality. For example, they showed that more successful counselors spend a longer time exploring solutions, while less successful counselors spend more time defining the problems.

Finally, the complexity and severity of help-seekers’ situations may lead to writer’s block in counselors. Although not directly related to the suicide prevention domain, Isbister et al [16] have designed a helper agent for human-human interaction. When a conversation lags, the agent suggests topics for the conversation pair to talk about and, thereby, the agent is generally able to make positive contributions to the chat.

In situations where counselors experience writer’s block, a straightforward solution would be to approach a senior colleague for help. These senior counselors can read along and describe in as much detail as necessary how they would respond to the help-seeker. However, this requires availability and time from a colleague, and this is not always possible. Responding quickly is important in life-threatening situations, and counselors cannot always wait for somebody to become available. We also suspect that an approach such as suggesting topics to keep a conversation going [16] or providing a conversation summary [12] would not be optimal in difficult situations where counselors have to de-escalate a suicide-related crisis. This paper, therefore, presents a system that uses natural language processing techniques to provide support for counselors in difficult chat conversations. The system recommends parts of similar, previous chat situations for the counselor to draw inspiration from, which might be able to reduce their writer’s block. This paper also evaluates the designed support system by comparing it with 1) written, general advice from a senior counselor and 2) receiving no additional help during chats. The system’s usability and utility, along with the validity of the algorithm used, were also examined.

## Methods

### Design

We used a within-subject design to evaluate the impact and usefulness of similar chat situations that could be used as inspiration. In the study, the counselor wrote a chat reply to a simulated chat that was interrupted as a difficult situation. The counselor took part in 3 simulations: 1) the counselor received parts of similar chats from a support system, 2) the counselor received written advice from an experienced counselor, and 3) the counselor received no additional help. A questionnaire was used to measure the support system’s usability. Finally, we evaluated the validity of the similarity of the generated chats by testing the algorithm in a small additional experiment with a within-subject design.

The current study received ethical approval from the TU Delft University research ethics committee (id: 688). Before starting the data collection, the experimental setup was also preregistered on the Open Science Framework [17].

### Recommender Support System

**Figure 1:**
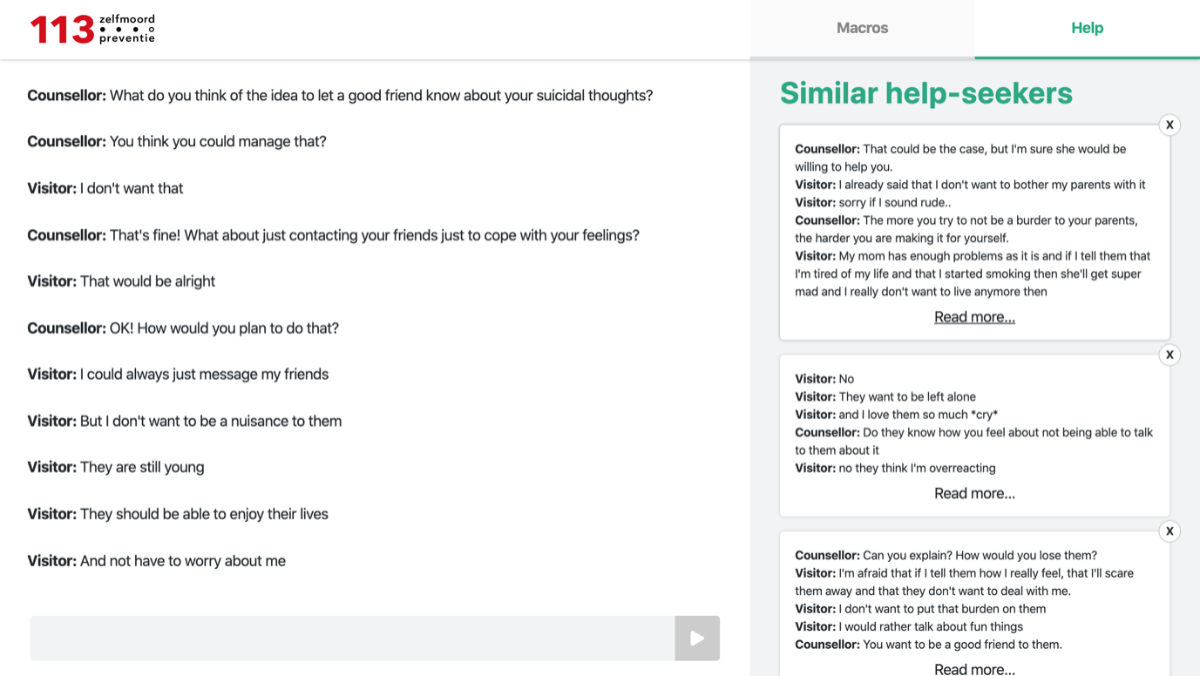
Interface support system (right). Content translated from Dutch.

For the study, we developed a system recommending the transcripts of similar previous chat conversations to a counselor based on the content of the counselor’s current chat conversation. Figure 1 shows a chat window on the left and the support system interface on the right. The support system shows the top 10 most similar chat messages, which the counselor could click to read them in their entirety.

A corpus of chat conversations between help-seekers and counselors was used to find similar previous chat situations. We used the corpus from 113 Suicide Prevention in the Netherlands. This corpus contained 7 months of chats spanning from March 2018 to September 2018. The chat data were first filtered, removing all chats that had less than 20 interactions. In total, we used 17,773 chats. Furthermore, any special symbols in the messages were cleaned, and capital letters were replaced by lowercase letters.

Because the chats each contained multiple problems, we used a sliding window algorithm to scan for relevant chat segments instead of comparing complete chats. This algorithm created sets of chat messages, starting with the first 5 messages. The next set removed the first message in the window and added the sixth message; this process was repeated to create every possible set of 5 subsequent messages in a chat. The sliding window algorithm was then used to create the chat segments for the entire corpus.

We used an embedding algorithm to compute the similarity. For each chat segment, an embedding was created using smooth inverse frequency [18], which takes a weighted average of the word embeddings for each word in the text of the window corresponding to the inverse of the frequency of the word in the corpus. This resulted in less meaningful words receiving a lower weight. To create word embeddings, Mikolov et al [19] developed an algorithm dubbed Word2Vec, improving previous methods [20]. The word embeddings we used were obtained from the COOSTO Word2Vec model [21], a model developed using Dutch social media and blog posts. A window of 5 messages resulted in 1,286,659 embeddings, which were stored alongside the corresponding chat and window positions.

When a counselor in an ongoing conversation requested similar chat conversations, a single smooth inverse frequency embedding was created using the same steps as with the corpus, except only the last 5 messages of the ongoing conversation were used. This embedding was then compared with the corpus embeddings through a cosine similarity. Ten windows with the highest similarity were recommended to the counselor.

### Difficult Chats

We used 6 chats for the experiment to cover several difficult situations: a situation where a help-seeker 1) was in a dangerous location and had withheld this from the counselor; 2) did not want to inform anybody in their environment of their suicidality because they felt like it would put a burden on others; 3) was afraid of people in their environment not understanding their problems; 4) tried to look for help but was not believed; 5) was excessively rude; and 6) had to contact a psychologist.

### Participants

Counselor and expert recruitment, as well as conducting the experiment, happened at 113 Suicide Prevention. In total, 24 counselors participated. On average, the participants’ age was 27 years old, and 79% were female. Only counselors who were interns, volunteers, or trainees were eligible to participate. Each counselor met all the components and conditions of the evaluation.

### Measures

The perceived utility was assessed with the following question: “How, in your opinion, did the extra information help you with coming up with your response?”. The counselors graded each support type on a fixed interval scale from –3 to 3, where –3 indicated the extra information was hindering, 0 indicated the information was neutral, and 3 indicated the information was useful.

To measure usability, the counselors were asked to fill out the System Usability Scale questionnaire [22]; this is a validated 10-item questionnaire with a 5-point scale ranging from “Strongly disagree” to “Strongly agree.”

To measure the validity of the algorithm, the counselors used a 7-point fixed interval scale to indicate how much they agreed with the following statement: “The problem in the matched chat is the same as the problem in the ongoing chat.” A score of 1 meant the counselor did not agree, whereas a score of 7 meant they did agree.

### Procedure

**Figure 2:**
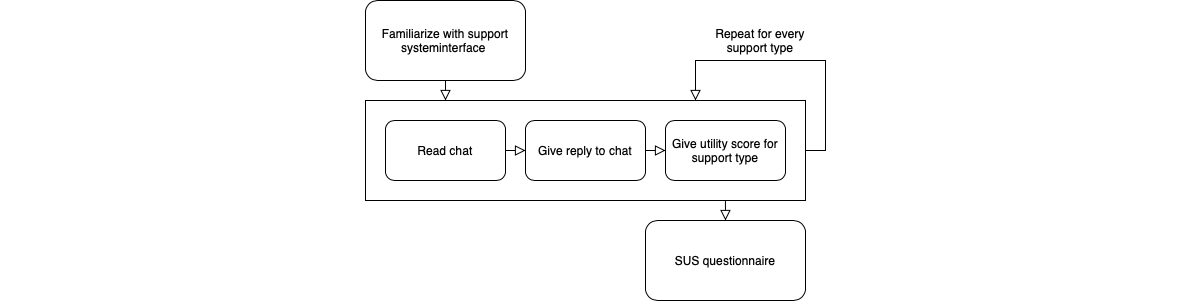
Procedure diagram of the first part of the experiment. SUS: System Usability Scale.

The counselors used a test environment with simulated chats. The experiment consisted of 2 parts. Figure 2 shows a diagram of the procedure for the first part. Before the experiment, the counselor had 5 minutes to explore and familiarize themself with the support system.

**Figure 3:**
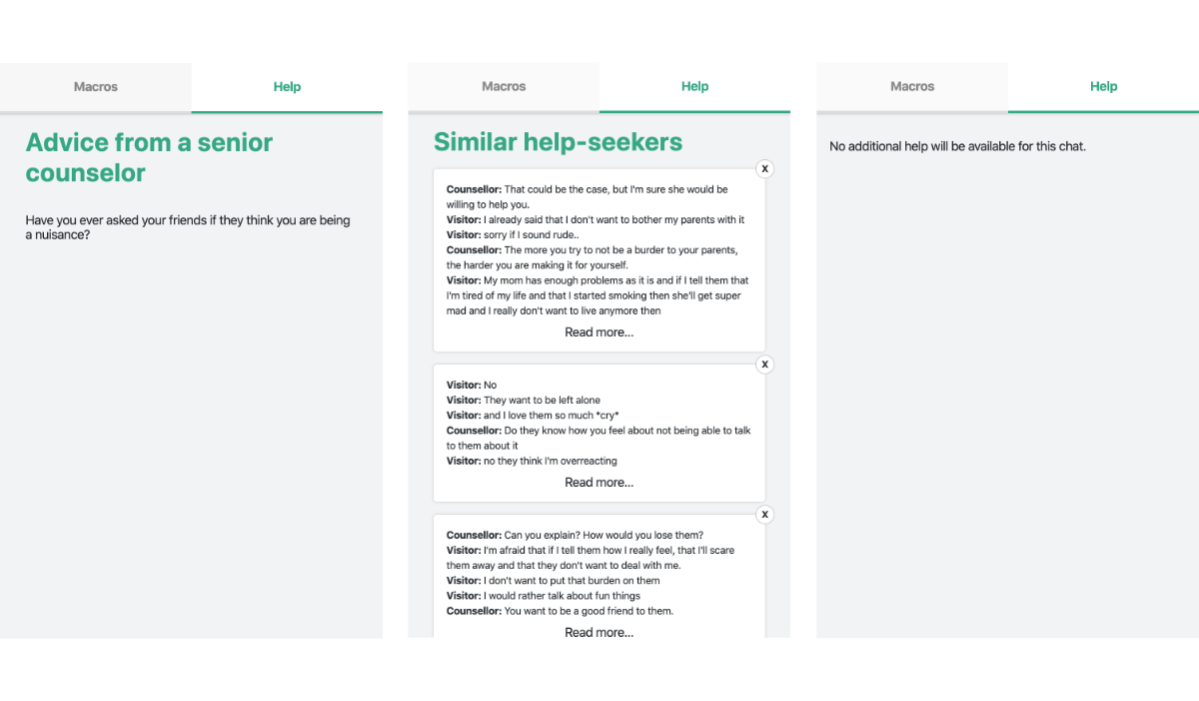
Conditions of the experiment: senior counselor written advice (left); support system (center); no additional help (right). Content translated from Dutch.

Part 1 consisted of a simulated environment where the counselor read and reacted to 3 simulated chats, one after the other. The support information was contained in an extra tab called “Help.” Figure 3 shows each support type. Each counselor had the same amount of time to read the chat. To simulate a real situation, each counselor had a 2-minute window to reply. The counselor could not access the support tab before the 2-minute timer started. Directly after the counselor submitted their reply to a chat, they were asked to rate the utility of the support type. These steps were repeated for each condition. Therefore, the participants reacted to 3 chats in total. The chats, support types, and combinations were counterbalanced for the 24 participants. This part ended with the System Usability Scale questionnaire being used to measure the usability of the support system.

**Figure 4:**
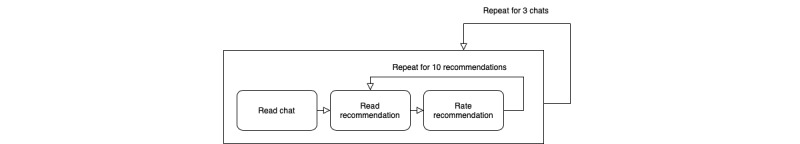
Procedure diagram of the second part of the experiment.

Part 2 recorded the measurements for evaluating the validity of the algorithm. Figure 4 shows a diagram of the procedure. The left side of the screen contained the transcript of an ongoing chat. The right side of the screen showed 10 chat segments. Half of these segments were randomly selected, and the other half was matched to the ongoing chat using the embedding algorithm. Below each of the segments was a fixed interval scale from 1 to 7 where the counselor rated the degree to which that chat segment related to the ongoing chat. To enhance generalization, the participants did this for the transcripts of 3 different ongoing chats. Therefore, in total, a participant rated 30 segments.

### Data Preparation

Eight experts labeled the reply of the counselor with the type of help (condition) that the expert assumed that the counselor had received. To prevent expert bias, each expert judged all the counselor responses. Furthermore, a reliability analysis for the items of the System Usability Scale questionnaire showed an acceptable level of consistency, with a Cronbach alpha of .89. Therefore, the System Usability Scale items were compiled into a single score.

### Analysis

The noticeable difference in counselor outputs was analyzed using generalized mixed-effects analyses [23] to predict the outcome variable support type based on the label the expert assigned to the counselor reply. The analyses were done by comparing 2 support type conditions at a time, thereby excluding the data from 1 of the 3 support type conditions. The models fitted on the remaining 2 conditions hence assumed a binomial distribution. Each model was compared with a null model that did not include an expert label as a fixed effect. Because the test was conducted 3 times, a Bonferroni correction [24] was used to set the significance threshold at .016. In addition, crossed random effects were used with random intercepts for the counselor and expert. Furthermore, for each support type, the utility ratings were analyzed using a one-sample *t* test to examine whether the rating deviated from the neutral zero score on the scale.

To examine the validity of the algorithm, a linear mixed-effects analysis was performed on the counselor’s rating of the similarity between the chat segment and the ongoing chat. As a two-level fixed effect the analysis included the recommendation method, that is, randomly selected versus selected by embedding algorithm. Furthermore, the ongoing chat was added as a three-level fixed variable because the quality of the suggestions was assumed to depend on the specific chat. As a random effect, the intercepts for counselors were used.

Anonymized data and R scripts are available online [25].

## Results

### Noticeable Difference in Counselor Output

**Figure 5:**
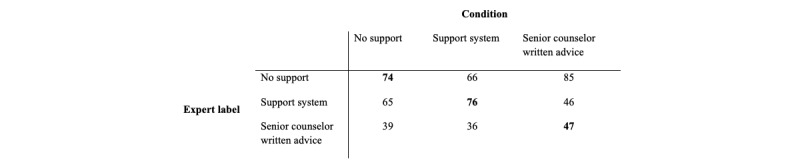
Confusion matrix for expert labeling of counselor responses.

Table 1 shows the effect of support type on the outcome measure of the expert label. The first row shows that the expert label significantly predicts the support type, when the data of no support condition was left out. In other words, the experts could tell the difference between replies given with the support system and replies with help from a senior counselor. Table 2 shows that when the expert labeled the counselor’s response as having received help from the support system, the counselor was 0.47 times less likely to have received the senior counselor support. This effect is further illustrated when looking at the confusion matrix of these conditions, as shown in Figure 5. However, no significant difference was found between the no support condition and any of the other conditions.

**Table 1:** Results of the comparison between null model and full models that included the expert label as a fixed effect to predict support type counselors had received when writing their reply (n=356).

Outcomes data included in analysis	χ2 (*df*)	*P* value
Support system and senior counselor written advice	11.31 (2)	.004
No support and support system	1.44 (2)	.49
No support and senior counselor written advice	4.78 (2)	.09

**Table 2:** Fixed effect of the expert label for the model of support system and senior counselor written advice.

Parameter	OR^a^	Standard error	z Value	*P* value
Intercept	1.29	0.16	1.54	.12
Support system	0.47	0.25	–3.04	.002
Senior counselor written advice	1.01	0.28	0.049	.96

^a^OR: odds ratio.

### Utility

The results of the utility ratings are shown in Table 3. The mean score of the support system was –0.21 (SD 2.26) and did not significantly deviate from 0, indicating that there was neither a hindering nor a helping effect experienced by the counselors. However, the mean utility score of the written advice from a senior counselor was 1.46 (SD 1.91) and significantly deviated from 0. This suggests that the written advice was perceived as helpful. It is noteworthy that the support system had a high variance, suggesting that the counselor’s opinion on the utility was divided.

**Table 3:** One-sample t test for counselor utility ratings per support types (n=24).

Support type	Mean (SD)	95% CI	*t*_df_	*P* value
Support system	–0.21 (2.26)	–0.84 to 0.43	–0.68_23_	.5
Counselor written advice	1.46 (1.91)	0.87 to 2.04	5.17_23_	<.001
No support	–0.21 (0.95)	–0.62 to 0.2	–1.04_23_	.31

### Usability

The mean score of the support system for the System Usability Scale questionnaire was 71, with a 95% confidence interval of 63-78. According to Bangor et al [26], this score can be classified as “good” based on an adjective rating scale.

### Validity of the Algorithm

How the chat segments were selected (randomly vs by the embedding algorithm) significantly predicted the rating counselors gave on the chat segment’s similarity to the ongoing chat, β=.30, t(7.66), *P*<.001. This means that counselors could tell the difference between the random chats and those generated by the support system. The suggestions from the algorithm increased the similarity rating given by counselors from an average of 2.35 to an average of 3.42 (difference of 1.07).

### Discussion and Conclusions

In the current study, we evaluated a prototype support system to assist chat counselors in suicide prevention helplines by providing inspiration from previous chats. The results show that counselors gave different answers depending on whether they received help from the support system or from a senior colleague. Upon inspection, the replies given by the counselors who received written advice from a senior colleague were, for the most part, copied directly and with little to no alterations made. Replies from counselors using the support system were more varied. This could be a possible explanation for the noticeable difference. However, we could not find a significant result for the no-help condition, which also had varied replies. Additionally, we observed that written advice from a senior counselor was given a significantly higher utility score than the other conditions; this suggests that the counselors value short actionable information that is highly accurate to the situation and that is given by someone with expertise. Gunaratne et al [27] have observed similar findings in their study on the effects of expert advice and social comparison on decision making for retirement savings; they showed that expert advice helped people make better decisions, whereas social comparison was seen as a useful mechanism to keep people from deviating too far from the mean and, hence, make safe decisions. However, both of these conditions outperformed a control condition where no additional information was provided.

The main contribution of our study is the idea of retrieving inspiration from a conversation corpus. Other support systems for chats [28-30] have used topics to assist the conversation. Compared with these methods, our approach for combating writer’s block in a counseling conversation is novel. Furthermore, an experimental design was used to compare this supportive technology with advice from a senior colleague, showing how the two differ.

Some limitations should be considered regarding the findings and their implications. We used chat transcripts of conversations with situations that previous counselors found difficult to evaluate. However, this might not cause writer’s block for every participant because not every counselor will have problems with the same situations. For writer’s block to occur naturally, the system would have to be tested in live chats. This was, however, not possible because of the ethical constraints of deploying an unevaluated system in a possible crisis situation. Furthermore, the specification, development, and evaluation were done in the context of counselors working at 113 Suicide Prevention in the Netherlands, with a limited number of counselors. The support system should also be tested in different helplines and with a larger sample size.

We have outlined two major directions for future work. First, the recommendation mechanism could be improved in different ways. This study, as well as other related works such as recommenders for creativity [31] and scientific writing [32], relies on topic modeling and bag-of-word models to find recommendations. Encoding text using attention-based models [33], such as BERT [34], have been shown to perform well on various natural language processing tasks, including semantic sentence similarity for conversation data [28]. These methods could be applied to improve the recommendations to find more relevant and similar examples, which we expect will increase the perceived utility. Additionally, curating the corpus can help denoise the dataset and improve the recommendations. This can also give counselors the knowledge that the information comes from a subset of quality chats, thereby acting on the persuasive principle of authority as outlined by Cialdini [29]. Lastly, there is also an opportunity to apply active learning methods by adding positive labels to the recommendations that the counselors interacted with or explicitly marked as useful [30].

Second, the findings show that the embedding algorithm found similar chats and that written advice from senior counselors had high utility. Compared to the Gunaratne et al study [27], the main difference to the setup of our study is that the social comparison condition provided information as an average; this indicates that refining the output of the support system recommendations to be more instructional could be a possible direction for improving the system. To combine both the extensive coverage of a chat corpus and the high utility of curated written advice, clustering could be used, that is, grouping similar chats together based on a similarity metric and curating the labels based on these clusters. Derrar [35] uses clustering to automate the annotation of customer service chat messages. A similar approach could be used to annotate the chat corpus to create a taxonomy of situations and advice, which then could emulate receiving written advice from a senior colleague. In other words, working together with experts, a set of advice could be formulated in advance for each specific situation. Next, a data-driven algorithm could be trained to classify chats according to categories of the taxonomy, consequently providing counselors with expert advice associated with the category and making the expert advice situation relevant. This approach would be most suitable for assisting counselors with frequently occurring tasks, as these would be the most likely cases to be included in the taxonomy. The focus of the support system might therefore shift from an inspiration source to a system that could reduce workload. Alternatively, the field of conversational information retrieval has explored multiple methods that could be applied to the task presented in this paper. For example, Qiu et al [36] combined both information retrieval methods and generation based models to create a chat bot trained using existing customer service chat logs. These techniques could potentially also be used to allow the system to generate proposal responses that counselors could consider using in their chats with help-seekers.

In conclusion, the current study shows a possible method to provide inspiration during chat counseling in a helpline for suicide prevention and how this supportive technology compares with human assistance. A support system may be a relief for counselors as they handle many cognitively difficult situations. In addition, supportive technology seems useful for helplines to better deal with busy periods, to provide a safety-net for junior counselors, and to help sustain counselors.
